# TRAP1 suppresses oral squamous cell carcinoma progression by reducing oxidative phosphorylation metabolism of Cancer-associated fibroblasts

**DOI:** 10.1186/s12885-021-09049-z

**Published:** 2021-12-14

**Authors:** Li Xiao, Qiannan Hu, Yanshuang Peng, Kaiyue Zheng, Ting Zhang, Lianjie Yang, Zhi Wang, Wanrong Tang, Jie Yu, Qian Xiao, Dandan Zhang, Weifang Zhang, Chanjuan He, Dengxun Wu, Yanyan Zheng, Ying Liu

**Affiliations:** 1 Department of Stomatology North Sichuan Medical College, Affiliated Hospital of North Sichuan Medical College, Nanchong, China; 2 Department of Stomatology, Nan Chong Central Hospital, Second Clinical Medical College of North Sichuan Medical College, Nanchong, China; 3 Guanghua School of Stomatology, Guangdong Provincial Key Laboratory of Stomatology, Stomatological Hospital, Sun Yat-Sen University, Guangzhou, China; 4 School of Basic Medical Sciences, North Sichuan Medical College, Nanchong, China

**Keywords:** Oral squamous cell carcinoma, Cancer associated fibroblasts, Oxidative phosphorylation, Proteomics, TRAP1

## Abstract

**Background:**

Glucose metabolism in cancer associated fibroblasts (CAFs) within the tumor microenvironment is a material and energy source for tumorigenesis and tumor development. However, the characteristics and important regulatory mechanisms of glucose metabolism in fibroblasts associated with oral squamous cell carcinoma (OSCC) are still unknown.

**Methods:**

We successfully isolated, cultured, purified and identified CAFs and normal fibroblasts (NFs). Cell culture, immunohistochemistry (IHC) and CCK8, flow cytometry, Seahorse XF Analyzer, MitoTracker assay, western blotting (WB), transmission electron microscope, Quantitative real-time PCR (qPCR), immunofluorescence (IF), and Label-free quantitative proteomics assay, animal xenograft model studies and statistical analysis were applied in this study.

**Results:**

We demonstrated that the proliferation activity of CAFs was significantly enhanced as compared to NFs, while the apoptosis rate was significantly decreased. CAFs in OSCC preferentially use oxidative phosphorylation (OXPHOS) rather than glycolysis. Moreover, CAFs showed stronger maximal respiration, a larger substantial mitochondrial spare respiratory capacity (SRC) and higher adenosine triphosphate (ATP) production capacity than NFs. The results of mitotracker green fluorescence staining showed that compared with NFs, CAFs exhibited stronger green fluorescence. The results of WB showed the expression level of Peroxisome proliferator-activated receptor γ coactivator-1α (PGC-1α) obviously increased in CAFs compared to NFs. These results confirmed that CAFs have greater mitochondrial activity and function than NFs. Furthermore, Label-free quantitative proteomics assays showed that both ATP synthase subunit O (ATP5O) and tumor necrosis factor receptor-associated protein 1 (TRAP1) are important differentially expressed proteins in the mitochondria of CAFs/NFs. Overexpression of TRAP1 in CAFs increased basal oxygen consumption rate (OCR), maximal respiration, ATP production and SRC. In vivo, overexpression TRAP1 expression in CAFs suppress tumor growth.

**Conclusion:**

Taken together, the results indicated that TRAP1 is an important regulatory molecule of CAFs glucose metabolism and promotes OSCC progression by regulating the OXPHOS of CAFs.

**Supplementary Information:**

The online version contains supplementary material available at 10.1186/s12885-021-09049-z.

## Introduction

Over the last few decades, it has become increasingly apparent that the occurrence and progression of tumors not only depend on the factors intrinsic to the tumor itself, but also require support from the tumor microenvironment (TME) [1]. Tumors are mixtures containing both neoplastic cells and stromal cells, which include tumor infiltrating immune cells, endothelial cells, neuronal cells, lymphatic cells, CAFs and the extracellular matrix (ECM) [2]. In OSCC, CAFs are one of the most abundant cell types found within the desmoplastic stroma [3]. Late- stage OSCC frequently consists of up to 80% CAFs [4]. Numerous studies have indicated that CAFs interact with cancer cells through various signaling molecules, which play an important role in cancer initiation, progression, and invasion [5–7].

Cells use two major pathways to produce ATP: glycolysis and mitochondrial respiration through OXPHOS. Conventional views suggest that malignant cells ferment glucose to make ATP, even in the presence of oxygen, which is a phenomenon known as the Warburg effect [8]. While early studies of tumors suggested a predominant role for glycolysis, more recent work has identified a role for mitochondria in metabolic reprogramming, emphasizing functions for TCA cycle intermediates (such as α-ketoglutarate and citrate) and respiration to support cellular proliferation and energetics [9, 10]. One of the most striking characteristics of cancer cells is their altered metabolism, which is adapted to support rapid proliferation by cross talk with the TME. Therefore, tumor cells within the microenvironment use different factors to activate nearby fibroblasts and control them, taking advantage of their metabolism according to the reverse Warburg effect [11]. However, the mechanism behind this phenomenon is still not completely understood. Further, as one of the most abundant components in the TME, the role of CAFs in TME metabolism **is** still unclear.

Mitochondria are the organelles responsible energy production, but they are also involved in carcinogenesis, cancer progression, and metastasis, where they play a role in altered energy metabolism in cancer cells. Some previous studies have shown that mitochondrial dysfunction in CAFs is attributed to stress in the TME, such as hypoxia and oxidative stress [12]. However, a recent study found that CAFs demonstrated significantly higher rates of oxidative phosphorylation than cancer cells do and that CAFs used lactate more efficiently than cancer cells [4]. Although this phenomenon is still not completely understood, it seems to implicate an increase in the mitochondrial function of CAFs, which contributes to tumor progression. Therefore, in the TME, the metabolic characteristics and mitochondrial function of fibroblasts associated with OSCC have not been elucidated. Overall, we aimed to study the metabolic characteristics and mitochondrial function of CAFs and the underlying mechanism behind these phenomena.

## Materials and methods

### Specimens

Fresh, sterile tissue specimens, including OSCC and para-carcinoma tissue were from North Sichuan Medical College in Sichuan, China. All specimens were collected after obtaining informed consent from patients. The pathological or normal state of the samples was confirmed by histopathological and clinical examination.

### Isolation, cultivation and purification of CAFs and NFs

After removing epithelium and adipose tissue, the remaining tissue was cut into pieces (1 mm × 1 mm × 1 mm). The tissues were cultured in DMEM (Gibco, USA) containing 10% fetal bovine serum, 100 IU/ml penicillin and 100 μg/ml streptomycin in an incubator at 37 °C with 5% CO_2_. The medium was changed every 2–3 days. When cells filled the bottom of the bottle, clumps of growing epithelial cells were removed by treatment with trypsin. The remaining cells were digested with trypsin for 2 min. Cultures at passages 3–4 were used for studies.

### Identification of CAFs through immunohistochemistry

The cells attached to the coverslips were fixed with 4% paraformaldehyde for 5 min. Staining procedures were conducted according to a previous study [13]. Both primary antibodies (anti-cytokeratin (CK), anti-vimentin and anti-α-smooth muscle (α-SMA)) and secondary antibodies were from Earthox (San Francisco, USA). Phosphate buffer saline (PBS) was used as a negative control instead of primary antibody. The cytoplasm stained with light brown or yellow brown cells was classified as positively stained cells.

### Analyses of cell proliferation

A cell counting kit (CCK8) (Yeasen, Shanghai, China) was used to evaluate the proliferation of CAFs and NFs. According to the manufacturer’s instructions, the cells were inoculated into a 96-well plate and then incubated at 37 °C for 24 h, 48 h, 72 h and 96 h. Then, 10 μl of CCK8 reagent was added to each well at the indicated time points and incubated for 1 h. Then, the absorbance was measured at a wavelength 450 nm.

### Analyses of cell apoptosis

An Annexin V-APC/7-AAD apoptosis kit (Liankebio, China) was used in this experiment. Per the manufacturer’s instruction, cells were washed twice with precooled PBS and then resuspended in 1X Binding Buffer at a concentration of 5 × 10^6 cells/ml. Annexin V-APC (5 μl) and 7-AAD (10 μl) were added to each tube and subsequently incubated for 5 min at room temperature in the dark. Then the cells were analyzed by flow cytometry.

### Measurement of glycolysis and OXPHOS in cells

CAFs and NFs were seeded in XFe 96-well microplates (12,000 cells/well) (Agilent Technologies, Sana Clara, USA). Cells were washed and incubated in base medium (Agilent Technologies) at 37 °C for 1 h. Then, extracellular acidification and oxygen consumption rates (extracellular acidification rate (ECAR) and oxygen consumption rate (OCR), respectively) were measured in XF media (nonbuffered RPMI 1640 containing 10 mM glucose, 2 mM L-glutamine, and 1 mM sodium pyruvate) under basal conditions and in response to 1 μM oligomycin, 1.5 μM fluorocarbonyl cyanide phenylhydrazone (FCCP), and 0.5 μM rotenone + 1 mM antimycin A by using the Seahorse XFe96 Analyzer (Agilent Technologies) according to the manufacturer’s instructions.

### Western blot analysis

Whole cell lysates were prepared in RIPA lysis buffer (CWBIO, China) supplemented with protease inhibitors. Equal amounts of proteins were loaded on an SDS-PAGE gels, and then were transferred onto PVDF membranes (Millipore, USA), which was followed by blocking for 60 mins at room temperature in 5% non-fat dry milk. Next, the membranes were incubated overnight at 4 °C with the following primary antibody: rabbit anti-human peroxisome proliferator-activated receptor γ coactivator-1α (PGC-1α) (HUABIO, China, 1:1000), rabbit anti-human transcription factor A mitochondrial (TFAM) (HUABIO, China, 1:1000), mouse anti-human cytochrome c oxidase (COX IV) (Earthox, USA, 1:1000), rabbit anti-human histone 3 (H3) (Abcam, USA, 1:1000) and mouse anti-human β-tubulin (Earthox, USA, 1:2000) overnight at 4 °C. Then, membranes were incubated secondary antibody for 1 h. The proteins were detected using the Chemiluminescence Kit (Millipore, USA). Densitometric analyses were performed using ImageJ.

### Mitochondrial mass determination

The mitochondrial mass was assessed using MitoTracker kit (Yeasen, Shanghai, China). Cells (5 × 10^4^/well) were plated onto 6-well culture dishes. After washing three times using PBS, the cells were incubated with 100 nM Mito Tracker for 40 min in the dark at 37 °C. The nuclei were stained with DAPI for 10 min. After washed three times using PBS, images of CAFs and NFs were captured under a confocal laser microscope.

### Isolation, purification and identification of mitochondria from CAFs and NFs

CAFs were generated from resected specimens from three OSCC patients undergoing curative surgery. Isolation and purification of mitochondria from CAFs and NFs were performed using a minute mitochondria isolation kit (Invent Biotechnologies, USA). According to the manufacturer’s instructions, all centrifugation steps were performed at 4 °C; briefly, cells were washed once with cold PBS and then were resuspended in 250 μl of buffer A. The cell suspension was incubated on ice for 10 min, transferred to a filter cartridge and then centrifuged at 14000 rpm for 30 s. The pellet was resuspended and centrifuged at 3000 rpm for 1 min. Next, the supernatant was transferred to a fresh 2.0 ml tube, and 400 μl of buffer B was added to the tube. The mixed suspension was centrifuged at 14000 rpm for 10 min. The supernatant was completely removed completely and the pellet was resuspended in 200 μl buffer B and centrifuged at 10000 rpm for 5 min. The pellet was washed once more and then was centrifuged at 16000 rpm for 15 min. The supernatant was discarded, and the pellet was saved.

Mitochondrial protein and whole cell lysates were prepared in RIPA lysis buffer (CWBIO, China) supplemented with protease inhibitors. The abundance of COX IV and H3 was compared between mitochondrial protein and whole cell lysates by loading equal amounts of proteins for Western blot analysis as previously outlined in detail. Transmission electron microscopy was used to ensure the quality of mitochondria. The purified mitochondria were fixed in 4% paraformaldehyde and 2.5% glutaraldehyde for 48 h, and washed with 0.1 M PBS buffer three times, and fixed for 1 h in 1% osmium tetroxide. Staining was performed overnight with 0.5% aqueous uranyl acetate. Then the samples were dehydrated in a graded series of ethanol (75, 85, 95, and 100%), embedded and stained with uranyl acetate/lead citrate. The sections were examined with a transmission electron microscope.

### Label-free quantitative proteomics analysis

#### After the mitochondria of CAFs and NFs were successfully extracted

Mitochondrial protein quantification was then performed by BCA. The purified mitochondria were washed twice in PBS, and lysed in RIPA lysis buffer (CWBIO, China) supplemented with protease inhibitors. The lysate was heated to 60 °C for 1 h in 0.05 M TCEP solution (Sigma, 646,547) and then was placed at room temperature away from light in 55 mM MMTS solution (Sigma, 208,795). The lysate was added to a 10 KDa ultrafiltration tube (Pall, OD 010C33) and centrifuged at 12000 g for 20 min. Next, 100 ul of UA (8 M urea pH 8.5, Amresco, 0568) solution was added to the lysate and then centrifuged at 12000 g for 20 min, twice. One hundred microliter 0.25 M TEAB solution was added to the lysate and then centrifuged at 12000 g for 20 min. The entire centrifugation process was repeated three times. Then, 2% trypsin (Promega, V5280) was added to the sample, and incubation was conducted at 37 degrees overnight. The next day, booster digestion was performed using an additional dose of trypsin. After digestion, the peptides were dissolved in sample solution (0.1% formic acid and 2% acetonitrile) and centrifuged at 13200 rpm for 4 min; then, mass spectrometry was used to identify the supernatant.

### Tissue immunohistochemistry and immunofluorescence

FFPE tissue specimens were deparaffinized in xylene and rehydrated through exposure to graded ethanol solutions. Antigen retrieval was performed by incubating the slides in Tris-EDTA buffer (pH 9) at 100 °C for 15 min. Then, 3% H_2_O was used to inactive endogenous peroxidase activity. Slides were incubated with a primary antibody overnight at 4 °C, a secondary antibody for 30 min at room temperature, and DAB Chromagen staining for 5 min. For immunofluorescence, slides were stained with fluorescently-labeled secondary antibodies (1:200; Thermo Fisher). Quantification of IHC results was performed by two experienced pathologists. Signal intensity was calculated according to the number of positive cells and the degree of intensity. Both the intensity (0 = absent, 1 = weak, 2 = moderate, and 3 = strong expression) and percentage of positive cells (0 = 0%, 1 = 5–10%, 2 = 11–20%, 3 = > 20%) were assessed, and the scores were multiplied. Scores of 0 to 2 were considered negative, and scores of 3 to 9 were considered positive.

### Lentiviral vector construction and transfection

TRAP1 overexpression plasmids were constructed with the following target sequences: forward: 5- AGGACGACTGTTCAGCACG-3′, and reverse:5′-CCGGGCAACAATGTCCAAAAG-3′. overexpression plasmids targeting TRAP1 and GFP were purchased from Gene Copoeia and viruses were produced using protocols from Gene-Copoeia (https://www.genecopoeia.com). According to the Gene-Copoeia protocol, lentiviral particles were generated by Lipofectamine-mediated transfection of lentiviral expressing plasmids, the packaging plasmid psPAX2, and the envelope plasmid pMD2.G into CAFs, and lentiviral particles were collected to transduce target cells. Cells were plated (2 × 10^5^/well) in 6-well plates and allowed to grow for 24 h until they reached 70% confluency. The cells were then transfected with diluted virus containing media with 8 μg/ml polybrene for 4 h; then, the medium was replaced with serum-enriched medium and the cells were cultured for an additional 72 h. Transfected cells were pooled and treated with puromycin for 1 week. Subsequently, the transfected cells were collected and processed for follow-up experiments.

### Quantitative real-time PCR (qPCR)

Total RNA from cells was extracted according to the manufacturer’s protocol (E.Z.N.A Total RNA Kit, Omega, USA), and total RNA was reverse transcribed to cDNA in strict accordance with the manufacturer’s instructions (PrimeScriptTM RT Reagent Kit, TaKaRa, Japan), which was then amplified with SYBR (Roche) by qPCR. The sequences of the primers were as follows: TRAP1 (Forward: 5- AGGACGACTGTTCAGCACG-3′, and Reverse:5′-CCGGGCAACAATGTCCAAAAG-3′); and GAPDH (Forward: 5′-GACTCATGACCACAGTCCATGC-3′; and reverse: 5′-AGAGGCAGGGATGATGTTCTG-3′). The relative levels of TRAP1 mRNA were normalized to GAPDH reference gene expression and calculated via the 2^-△△CT^ method.

### Tumor xenografts

HSC3 cell line used in the tumor model were purchased from the ATCC. Six weeks-Balb/c-nu mice were purchased from GemPharmatech Co., Ltd. For the subcutaneous models, 5 mice were randomized in each experimental group. HSC3 cells and human CAFs that overexpression of TRAP1(sh-TRAP1) or other control cells (1:1, 1 × 106 cells in 100 μL of DMEM) were inoculated on the right flank of Balb/c-nu mice. Tumor length (L) and width (W) were measured with calipers every 2 days. The tumor volume was calculated by the formula: (L × W2)/2. At 22 days, the mice were euthanized and tumors were dissected.

### Statistical analysis

All statistical analyses were performed using GraphPad Prism 7.0 and Stata/MP 14.0. A two-sided *p* < 0.05 was considered statistically significant and is represented in figures as *p < 0.05, **p < 0.01, ***p < 0.001, and ****p < 0.0001. In addition, n.s in the figures represents no significant difference.

## Results

### Proliferation and apoptosis of CAFs

**Fig. 1:**
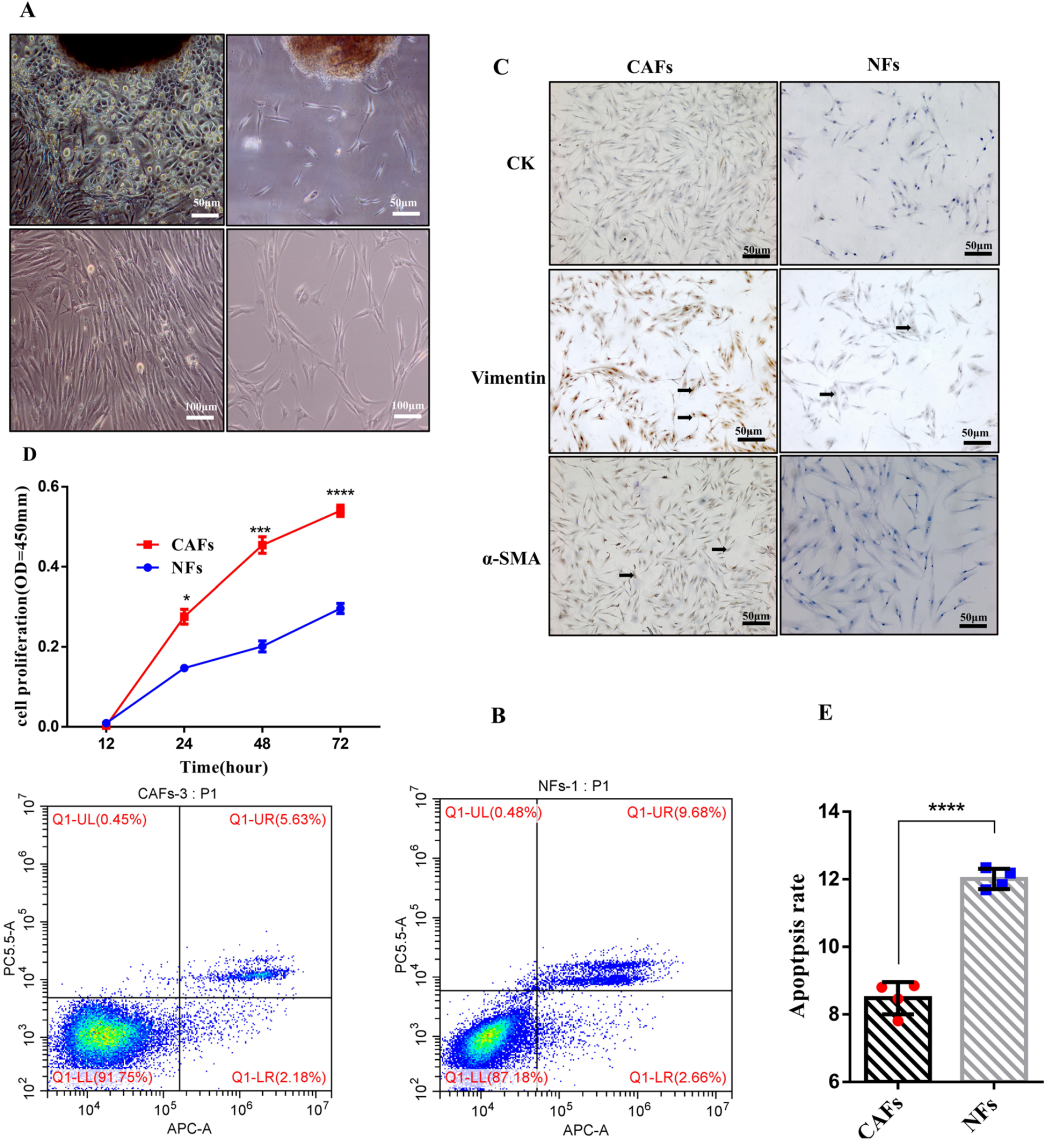
The proliferation and apoptosis ability of CAFs and NFs. **A** Comparison of the primary CAFs and NFs (phase-contrast microscope, × 50). **B** Comparison of the purified CAFs and NFs at passage 3 (phase-contrast microscope, × 100). **C** CAFs at passage 3, positive staining with α-SMA and Vimentin, negative staining with CK (× 50). **D** CAFs and NFs seeded in growth medium were enumerated at 12 h, 24 h, 48 h and 72 h. Data are presented as the mean values ± SD of three independent experiments. Statistically significant relationships are indicated by *p < 0.05, *** < 0.01 and ****p < 0.0001 (compared with NFs). **E** The flow cytometry analysis was used to examine apoptosis of CAFs and NFs, UR was considered as late apoptosis, and LR was considered as early apoptosis, respectively. The apoptosis rate is the sum of early apoptosis rate and late apoptosis rate. Data are presented as the mean values ± SD of three independent experiments. Statistically significant relationships are indicated by ****p < 0.0001 (compared with NFs)

To obtain passage three purified oral CAFs and NFs, we separated, cultivated and identified oral CAFs and NFs by tissue culture and trypsinization. As shown in Fig. 1A-B, the morphological characteristics of the CAFs changed significantly compared to those of the NFs. As shown in Fig. 1C, CAFs showed positive staining for α-SMA and vimentin and no staining for CK. In contrast, NFs showed positive staining for vimentin, and no staining for α-SMA and CK.

The cell proliferation rates of CAFs and NFs at 24, 48, 72, and 96 h are shown Fig. 1D; with the same culture conditions, number of seeded cells and observation time, we found that the proliferation speed of the CAFs was significantly higher than that of the NFs. To further investigate whether the increased cell proliferation of CAFs was due to a reduction in cell apoptosis, we used flow cytometry analyses to evaluate CAFs and NFs. We found that the proportion of CAFs in early apoptosis and late apoptosis was smaller than that of NFs, and the differences between their proportions were statistically significant (*P* < 0.0001) (Fig. 1E). Our study results suggested that CAFs acquired growth speed, proliferation and viability that were greater than those of NFs.

### CAFs demonstrate stronger mitochondrial respiratory capacity than NFs

**Fig. 2:**
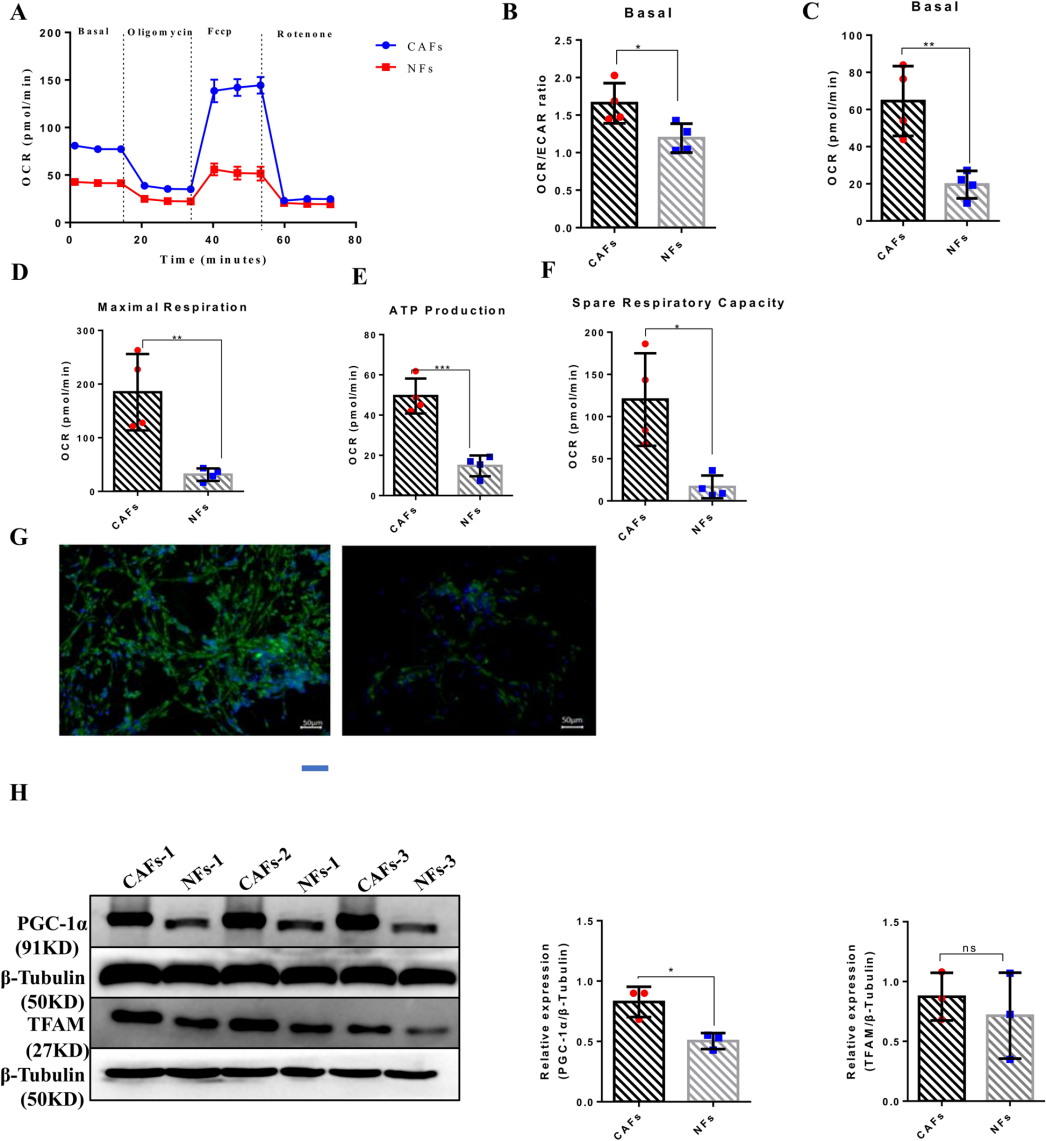
CAFs demonstrate stronger mitochondrial respiratory capacity than NFs. **A** Metabolism capacity of four patients derived CAFs and NFs were assessed by Seahorse flux analyzer. Data are representative of four independent experiments. **B-C** O2 consumption rates (OCR) and extracellular acidification rate (ECAR) were measured in real time under basal conditions. Basal OCR, *p < 0.05 (B) and OCR/ECR ratio **p < 0.01 (C)for CAFs versus NFs. Data are representative of four independent experiments. **D** After FCCP injection, Maximal Respiration of CAFs and NFs were assessed. **p < 0.01 for CAFs versus NFs. Data are representative of four independent experiments. **E** ATP production of CAFs and NFs were measured by Seahorse flux analyzer. ***p < 0.005 for CAFs versus NFs. Data are representative of four independent experiments. **F** Spare respiratory capacity (maximal respiration minus basal respiration) was assessed by Seahorse flux analyzer, *p < 0.05 for CAFs versus NFs. Data are representative of four independent experiments. **G** Confocal images show CAFs and NFs stained with Mitotracker (green) and nucleu (blue); scale bars represent 50 μm. **H** Western blotting was employed to examine the expression levels of PGC-1α and TFAM in CAF and NFs. Data are presented as mean values ± SD of three independent experiments, and n.s represents no significant difference. Statistically significant relationships are indicated by *p < 0.05

To determine whether the inhibition of apoptosis and rapid cell growth was related to the metabolic pattern and abilities, we assessed the ECAR indicator of glycolysis, and the OCR indicator of OXPHOS, in a basal state and after the addition of oligomycin (to block ATP synthesis), FCCP (to uncouple ATP synthesis from the electron transport chain, ETC), and rotenone and antimycin A (to block complexes I and III of the ETC, respectively) (Fig. 2A). In a basal state, CAFs possessed a higher basal OCR and OCR/ECAR ratio than NFs (Fig. 2B-C). This indicates that CAFs preferentially use OXPHOS rather than glycolysis. After FCCP injection, CAFs showed stronger maximal respiration than NFs (Fig. 2D). Following treatment with rotenone and antimycin A, the results showed that CAFs demonstrated a significantly higher ATP production capacity (Fig. 2E). Similarly, CAFs demonstrated a larger substantial mitochondrial spare respiratory capacity (SRC, maximal respiration minus basal respiration) than NFs (Fig. 2F). Together these data suggest that in the TME, CAFs produce ATP to adapt to rapid proliferation through OXPHOS.

To further determine whether CAFs have stronger mitochondrial function than NFs, we detected the mitochondrial activity of CAFs and NFs through MitoTracker green fluorescence staining. The results showed that compared with NFs, CAFs exhibited stronger green fluorescence (Fig. 2G), which further confirmed that CAFs have greater mitochondrial activity and function than NFs. PGC-1α, which is a well-known regulator of mitochondrial OXPHOS, enhances the transcription of TFAM, an important transcription factor in mitochondrial biogenesis and OXPHOS [14]. Therefore, to further assess the enhanced mitochondrial function and content of CAFs, we detected the expression levels of PGC-1α and TFAM in CAFs and NFs respectively through WB. The results showed that the expression level of PGC-1α obviously increased in CAFs compared to NFs (*p* < 0.05); further, and TFAM was slightly increased in CAFs compared to NFs, but there was no statistical significance (Fig. 2H). This suggests that CAFs preferentially use OXPHOS to generate energy, which is consistent with our previous results, and shows that CAFs have higher OCR/ ECAR ratios than NFs.

### Label-free proteomics reveals that proteins mainly involved in metabolic pathways are differentially expressed in the mitochondria of CAFs and NFs

**Fig. 3:**
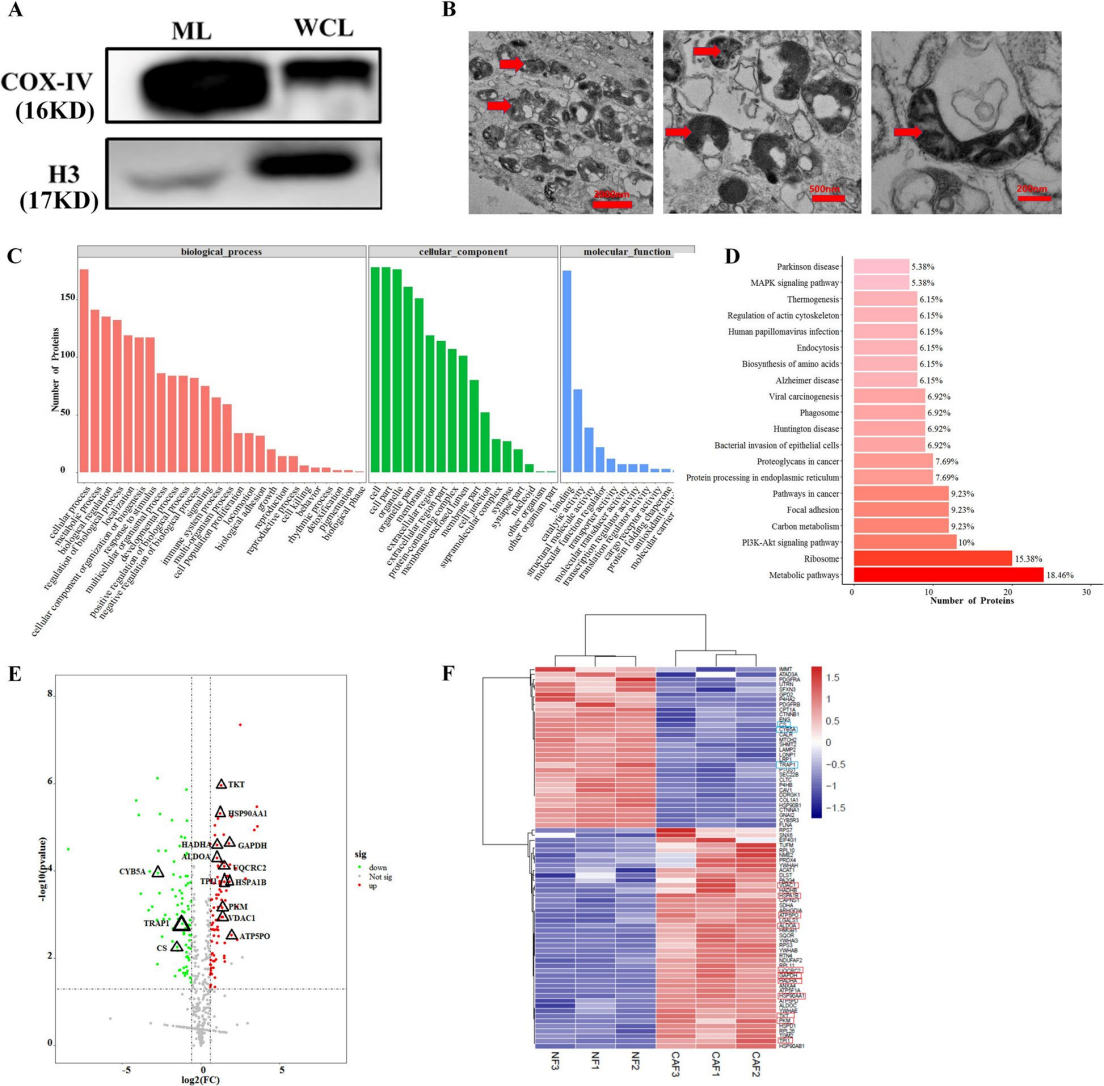
Label-free proteomics reveals that proteins mainly involved in metabolic pathways are differentially expressed in the mitochondria of CAFs and NFs. **A** Contamination and abundance of mitochondria preparations obtained by kit protocol was assessed by Western blotting. ML, mitochondrial lysate; WCL, whole cell lysate; **B** Integrity of purified mitochondria preparations were assessed by transmission electron microscopy. The image showed well- preserved mitochondria with electrondense matrix and cristae (red arrowheads). **C** Gene ontology (GO) annotation of differential proteins (CAFs/NFs) in mitochondria. **D** Kyoto Encyclopedia of Genes and Genomes (KEGG) annotation of differential proteins (CAFs/NFs) in mitochondria. **E** Volcano plot of a total of 183 differential protein in mitochondria (CAFs/NFs). The − log10 (*P* value) was plotted against the log2 (FC), FC: CAFs/NFs. The red dots represented proteins up-regulated in mitochondria (CAFs/NFs), green dots corresponded to proteins down-regulated in mitochondria (CAFs/NFs). **F** Heatmap of a total of 76 differential protein (CAFs/NFs) related to mitochondria. **G** Immunofluorescence and Immunohistochemistry analysis of ATP5O and TRAP1 expression in CAFs of OSCC and para-OSCC tissue. Data are presented as mean ± SD of three independent experiments, and n.s represents no significant difference. Statistically significant relationships are indicated by *p < 0.05

To identify molecular differences between mitochondria derived from CAFs and NFs, we successfully isolated, purified and characterized the mitochondria of CAFs and NFs for proteomic analyses. COX IV, which is the last enzyme in the mitochondrial electron transport chain, is a marker of mitochondria and is constitutively expressed mitochondria. H3 is a marker of the cell nucleus and is constitutively expressed in the nucleus. Therefore, equal protein amounts of whole cell lysates were assessed as controls to confirm the enrichment of mitochondria in the isolated fractions. The results showed low contamination, as indicate by low abundance H3 and high abundance of COX IV in the isolated mitochondria (Fig. 3A). In addition, using transmission electron microscopy, purified mitochondria quality was assessed, and the separated mitochondrial fraction was confirmed by the accumulation of round mitochondria with well-preserved mitochondria with an electron-dense matrix and cristae (Fig. 3B).

Next, a label-free LC-MS/MS proteomic approach was applied to analyze the differentially expressed proteins in mitochondria between CAFs and NFs. In total, 183 differentially expressed proteins were identified in mitochondria between CAFs and NFs of three OSCC patients (Supplementary Table S1); statistical analysis showed that 95 proteins were upregulated (≥1.5-fold change, *p* < 0.05) and 88 proteins were downregulated (≤0.67-fold change, *p* < 0.05). All 183 differentially expressed proteins were classified using the Gene Ontology (GO: http://www.geneontology.org) database according to biological process, cellular components and biological pathways (Supplementary Table S2). The data showed that metabolic processes were significantly enriched in the biological processes category (Fig. 3C). Similar results were observed after KEGG pathway analysis. KEGG analysis showed that the identified proteins were mainly involved in metabolic pathways (18.46%) (Fig. 3D). To further identify the target proteins related to metabolism, volcano plots and maps were generated, and they illustrate a total of 76 differentially expressed proteins related to mitochondria (Fig. 3E-F). Furthermore, OXPHOS-related proteins were identified by using Reactome databases. The results showed that 26 differentially expressed proteins were tightly associated with OXPHOS. Among the 26 differentially expressed proteins, 10 proteins positively correlated with OXPHOS were significantly upregulated (≥2-fold change, *p* < 0.05), 2 proteins down-regulated, and 1 protein that was downregulated was negatively correlated with OXPHOS (Supplementary Table S1). To confirm the proteomic results, we assessed ATP5O and TRAP1 expression in 16 OSCC species and adjacent mucosa through IHC and IF. To detect the expression of ATP5O and TRAP1 in CAFs of OSCC and NFs of para-cancer tissues, CAF-specific-α-SMA expression in OSCC and NF-specific vimentin expression in para-carcinoma tissue were also determined. The coexpression of ATP5O and TRAP1 with vimentin or α-SMA was examined using immunofluorescence double staining. The results showed that the expression level of ATP5O was significantly increased in CAFs compared to NFs, while the TRAP1 results revealed an opposite trend (Fig. 3G). The results are consistent with those of the proteomics. ATP5O is pivotal component of the mitochondrial membrane ATP synthase produces ATP from ADP in the presence of a proton gradient across the membrane which is generated by electron transport complexes of the respiratory chain [15, 16]. Since ATP5O is directly involved in ATP synthesis, it may belong to the terminal effector molecule family that is involved in the abnormal glucose metabolism regulation mechanism of CAFs. The molecular chaperone TRAP1, which is the mitochondrial isoform of cytosolic heat shock protein (HSP90), remains poorly understood with respect to its pivotal role in the regulation of mitochondrial metabolism [17]. Recent research has found that its role as an oncogene or a tumor suppressor, depends on the metabolic features of the specific tumor [18, 19]. However, the associations between OSCC glucose metabolism and TRAP1 are not clear. Therefore, we wanted to further analyze the role of TRAP1 in regulating glucose metabolism in OSCC.

### TRAP1 regulated the metabolism of CAFs by reducing OXPHOS

**Fig. 4:**
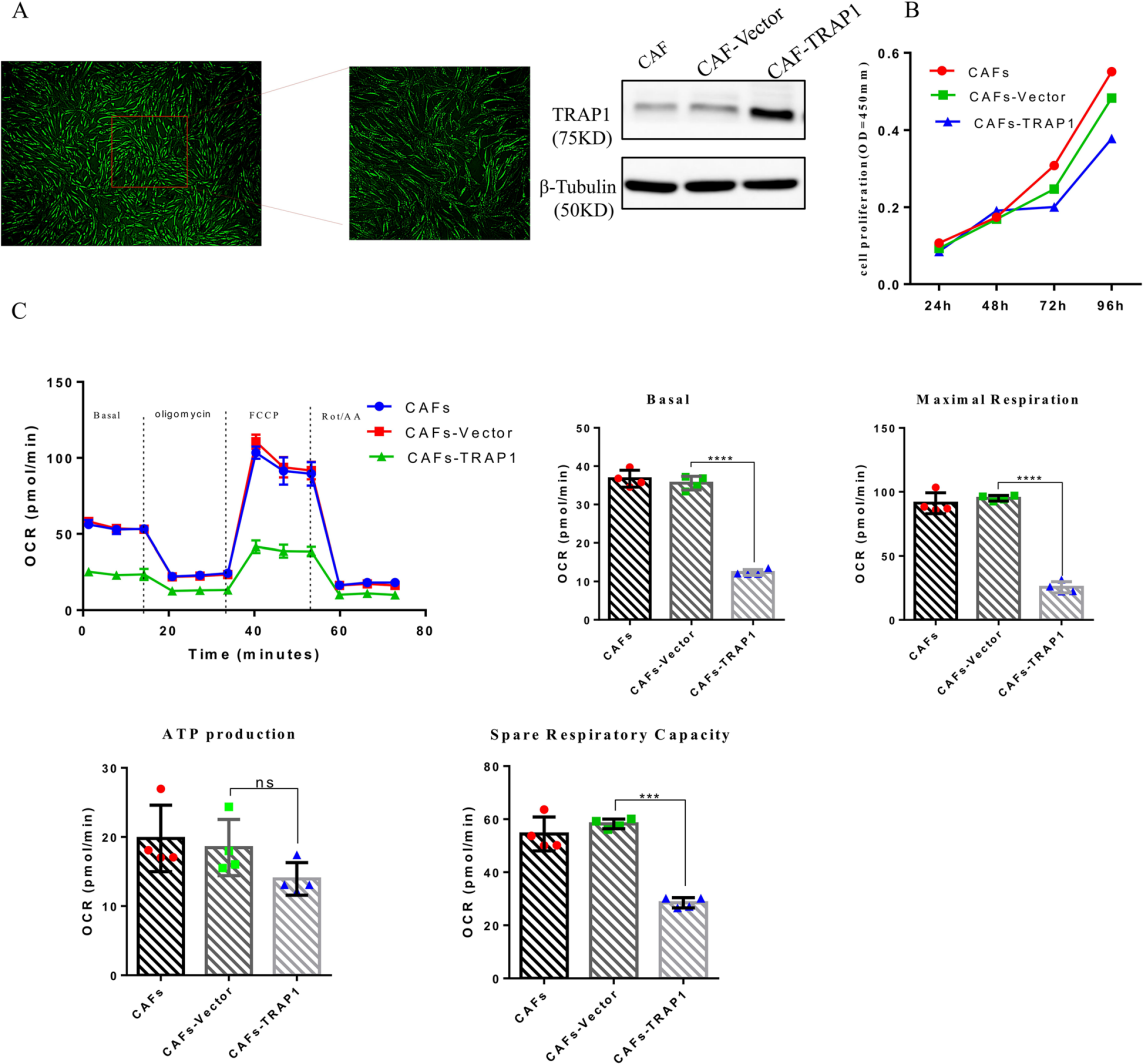
TRAP1 regulated the metabolism of CAFs by reducing OXPHOS. **A** TRAP1-GFP CAFs were verified with fluorescence microscopy; Efficiency of TRAP1 overexpression was confirmed using Western blotting. **B** TRAP1 overexpression CAFs and control cells seeded in growth medium were enumerated at 24 h, 48 h, 72 h and 96 h. **C** Basal OCR, ATP production, maximal respiration and SRC were assessed in CAFs after TRAP1 overexpression and vector transfected cells were used as controls. Data are presented as mean ± SD of three independent experiments, and n.s represents no significant difference. Statistically significant relationships are indicated by *p < 0.05, ***p < 0.005 and ****p < 0.0001

The role of TRAP1 in metabolic reprogramming is also controversial: its ability to enhance or suppress the switch from oxidative phosphorylation to aerobic glycolysis, known as the Warburg effect, seems to be context-dependent and as it differs in tumors [20]. In our results, mitochondrial proteomics analysis revealed that TRAP1 expression was decreased in CAFs compared to NFs, which was also confirmed in OSCC samples. TRAP1 expression is low in CAFs, which may be related to cell proliferation and glucose metabolism. It is well known that tumor glucose metabolism is closely related to tumor growth in vivo and in vitro. Thus, we hypothesized that increased OXPHOS of CAFs in the TME was caused by decreased TRAP1 expression. To verify this hypothesis, we established TRAP1 overexpression clones using CAFs as host cells, and their successful establishment was verified by WB (Fig. 4A). We found that TRAP1 overexpression significantly attenuated the proliferative ability of CAFs. Further, overexpression of TRAP1 in CAFs decreased basal OCR, which indicated that TRAP1 can reduce basic oxidative phosphorylation of CAFs. Overexpression of TRAP1 in CAFs enhanced maximal respiration and ATP production, which can provide more energy and material sources for cell tumor growth. Moreover, overexpression of TRAP1 in CAFs decreased SRC compared to that of vector cells, which increased the viability of tumor cells (Fig. 4C). These results suggested that TRAP1 can regulate the metabolism of CAFs by reducing OXPHOS.

### Overexpression TRAP1 expression in CAFs suppress tumor growth in vivo

**Fig. 5:**
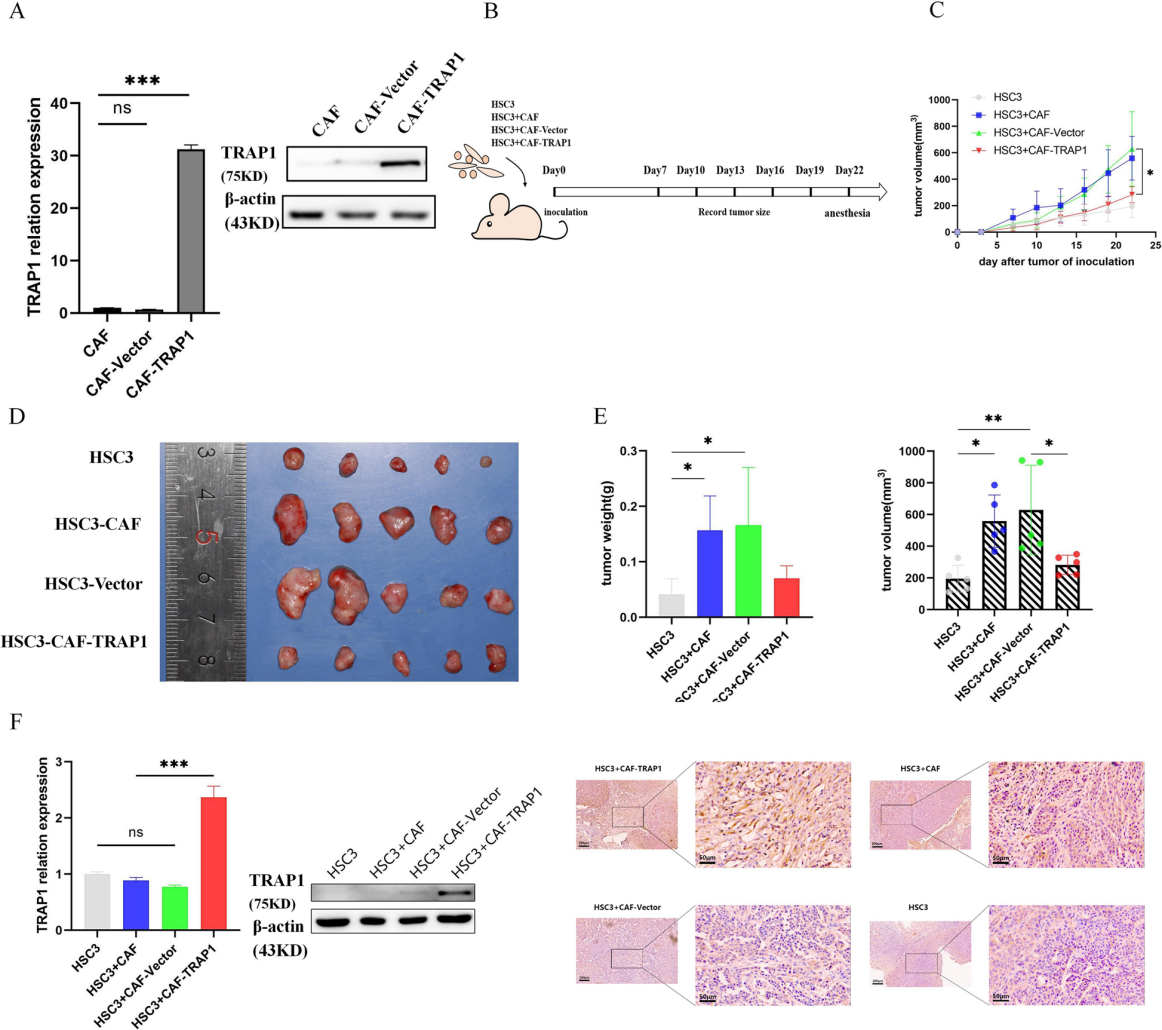
Overexpression TRAP1 expression in CAFs suppress tumor growth in vivo. **A** TRAP1 overexpression was confirmed using RT-PCR and Western blotting. **B** experimental timeline: HSC3 cells (1.0 × 106 cells/mouse) and conditioned CAF (1.0 × 106 cells/mouse) as indicated were inoculated to Balb/C-nu mice subcutaneously. **C** Tumor growth curves in control and experiment groups were measured as tumor volume over time. **D** Differences in tumor volume between control and treatment groups were analyzed statistically as described in the methods. *p < 0.05. *p* values calculated using two-way ANOVA with Tukey for multiple comparison. **E** Mice were sacrificed at day 22 after tumor cell injection. Photographs of tumor in nude mice. Differences in tumor volume and weight between control and treatment groups were determined by two-way ANOVA with *p* < 0.05 as being statistically significant. **F** Western blotting and Immunohistochemistry were employed to examine the expression levels of TRAP1 in four in vivo xenograft models

Given the findings of TRAP1 inhibition on mitochondrial energy metabolism in CAFs, we sought to determine the effect of TRAP1 in vivo. TRAP1 overexpression was confirmed using qPCR and WB (Fig. 5A). Four in vivo xenograft models were constructed to implement different combinations of human oral cancer cells and fibroblasts: HSC3, HSC3 + CAF, HSC3 + CAF-Vector, HSC3 + CAF-TRAP1(Fig. 5B). OSCC rapidly proliferates in the presence of CAFs, with a two-fold increase observed (Fig. 5C). Consistent with previous studies [4, 21], we observed CAF and OSCC xenograft tumors grow at a faster rate than OSCC alone. OSCC proliferation induced by CAFs-TRAP1 was significantly decreased compared with CAF-vector (Fig. 5C). Mice were sacrificed at day 22 after tumor cell injection (Fig. 5D). The tumor volume was smaller in the HSC3 + CAF-TRAP1 group than in the HSC3 + CAF-Vector group (Fig. 5E). To further confirm the above study results, the expression levels of TRAP1 were detected in four in vivo xenograft models by qPCR, WB and IHC. The IHC results show that expression of TRAP1 was increased markedly in the HSC3 + CAF-TRAP1 group compared with HSC3 + CAF-Vector group; expression Of TRAP1 was increased slightly in the HSC3 + CAF group compared with HSC3 group. These results of studies suggest that TRAP1 high expression was stable after CAFS transfection and tumor growth in vivo was closely related to the high expression of TRAP1 (Fig. 5F). These findings demonstrated that the overexpression TRAP1 in CAFs significantly suppressed tumor growth in vivo.

## Discussion

In recent years, complex tumor–microenvironment interactions have been recognized as important contributors to the progression and outcome of cancer. A previous study reported that cancer cells activate CAFs and regulate the CAF phenotype, including the expression of CAF markers, proliferation and apoptosis, through a variety of different pathways; in these ways, CAF regulation contributes to tumor growth and invasion [22]. Consistent with previous studies, the results indicated that CAFs highly expressed α-SMA, which is an important marker of CAF activation. Moreover, we found in this study that CAFs demonstrated stronger proliferation ability and weaker tendency for apoptosis than NFs. Additionally, a large portion of late-stage cancer tumors consist of CAFs, and activated CAFs regulate cancer progression via their active secretome, which includes growth factors and the extracellular matrix [23]. This creates crosstalk, impacting cancer metabolism, cancer immunity and drug resistance, where both cell types support each other to enhance proliferation, migration, and progression of cancer [24].

In recent years, it has been recognized that the progression of cancer cells is associated with altered energy metabolism in the TME, and as cancer progresses, the metabolic state of CAFs also coevolves—transitioning from a low to high metabolic state [25]. However, little is known about the metabolic switch of CAFs and the underlying mechanism. Therefore, our results revealed found that unlike cancer cells, CAFs produced ATP through mitochondrial OXPHOS. Additionally, CAFs have stronger OXPHOS ability, including maximal respiration, ATP production and SRC than NFs. The SRC is the extra capacity available in cells to produce energy in response to increased stress or work and so it is associated with cellular survival [26]. Our study hinted that CAFs with an enhanced SRC contributed to the progression of cancer in response to stresses to the TME, such as oxidative stress and low nutrients. The PGC-1α /TFAM axis plays a critical role in energy metabolism and mitochondrial biogenesis, and the members of this axis are expressed at high levels in cancer as a result of the abundance of mitochondria, active oxidative metabolism and response to increased energy needs [27]. To further confirm our results, we quantified the expression levels of PGC-1α and TFAM and found that the expression levels of PGC-1α and TFAM were markedly increased in CAFs compared to NFs. In addition, Mitotracker staining suggested that the mitochondrial mass of CAFs was increased compared to that of NFs, contributing to enhanced OXPHOS. This correlates with our finding that CAFs have stronger OXPHOS ability than NFs. Together our data indicated that CAFs have greater mitochondrial mass than NFs, which underlies their enhanced OXPHOS.

Indeed, emerging evidence shows that in highly aggressive tumors, mitochondrial energy pathways are reprogrammed to meet high energy demand, providing better utilization of available fuels and macromolecular synthesis for rapid cell division and migration [28–30]. We explored the significance of TRAP1 as a mediator of tumor metabolic programming in the stromal compartment of OSCC and a marker of tumor progression. To elucidate the mechanism of enhanced OXPHOS of CAFs in the TME, we performed differential proteomics identification to compare CAF and NF mitochondria. Proteomic analysis revealed that TRAP1 expression was downregulated along with 26 differentially expressed proteins associated with OXPHOS. TRAP1 is the only mitochondrial member of the HSP90 family that regulates a metabolic switch between mitochondrial respiration and aerobic glycolysis [31]. TRAP1 deficiency is associated with increased mitochondrial respiration and decreased glycolysis [32]. Consistent with our finding that TRAP1 overexpression inhibited CAF proliferation and mitochondrial respiration, previous studies have shown that TRAP1 promotes neoplastic growth by binding to and inhibiting succinate dehydrogenase complex II of the respiratory chain [33]. TRAP1 not only regulated tumor metabolism through mitochondria, but also regulated glutamate metabolism, which plays an important role in tumor therapy [34].

We further demonstrated that in vivo, TRAP1 overexpression in CAFs can promote the growth and progression of OSCC. TRAP1 has been reported to inhibit progression of numerous cancers, and we demonstrate that overexpression TRAP1 in CAFs reduces OXPHOS, proliferation and progression of OSCC in vivo and intro. These findings provide a mechanistic clue to explain the metabolic switch of CAFs and identify TRAP1 as a promising antineoplastic target in the TME. The Ras/ERK signaling pathway participates in the metabolic reprogramming that allows tumor cells to thrive under the conditions of limited oxygen supply that they encounter during tumor growth and progression [35]. In the mitochondrial matrix of neurofibromin-deficient cells, which exhibit enhanced glycolysis and decreased respiration in a Ras/ERK-dependent way, active ERK1/2 interacts with TRAP1 and SDH in mitochondria of neurofibromin-deficient cells and that ERK-dependent phosphorylation of specific residues on TRAP1 has a key role in their tumorigenicity [36]. Referring to the mentioned and our relevant works, our further work should clarify whether TRAP1 prompts a pro-neoplastic metabolic switch via regulation of transcription factors or epigenetic changes.

## Conclusion

In summary, our data suggest that OXPHOS is enhanced in CAFs in the TME in response to TRAP1 deficiency in mitochondria which is associated with changes in basal respiration, ATP production, maximal respiration and SRC, and Overexpression of TRAP1 in CAFs promotes tumor progression in vivo; these changes contribute to the progression of OSCC, making OXPHOS inhibition an attractive therapeutic strategy for targeting both tumor cells and the surrounding stroma. More studies are needed to provide important insights into the mechanism by which TRAP1 regulates CAFs metabolism in TME, which promotes the mutual utilization and coevolution between tumors and TME.

## Supplementary Information

**Additional file 1: Table S1.** Enrichment of cellular components, biological processes, and biological pathways related to mitochondria in differential proteins (CAFs/NFs).

**Additional file 2: Table 2.** Identified proteins associated with OXPHOS in different proteins of mitochondria (CAFs/NFs).

## Data Availability

All data generated or analyzed during this study are included in this published article (and its supplementary information files).

## Abbreviations

CAFs: Cancer associated fibroblasts
OSCC: Oral squamous cell carcinoma
NFs: Normal fibroblasts
IHC: Immunohistochemistry
WB: Western blotting
IF: Immunofluorescence
TRAP1: Tumor necrosis factor receptor-associated protein 1
ATP: Adenosine Triphosphate
OXPHOS: Oxidative Phosphorylation
ATP5O: ATP synthase subunit O
TME: Tumor Microenvironment
ECM: Extracellular Matrix
α-SMA: α-smooth muscle
CK: Cytokeratin
ECAR: Extracellular Acidification Rate
OCR: Oxygen Consumption Rate
FCCP: Fluorocarbonyl Cyanide Phenylhydrazone
PGC-1α: Peroxisome proliferator-activated receptor γ coactivator-1α
TFAM: Transcription Factor A Mitochondrial
COX IV: Cytochrome c oxidase
H3: Histone 3
SRC: Spare Respiratory Capacity
HSP90: Heat Shock Protein 90
