# Correction to: TRAP1 suppresses oral squamous cell carcinoma progression by reducing oxidative phosphorylation metabolism of Cancer-associated fibroblasts

**DOI:** 10.1186/s12885-021-09114-7

**Published:** 2021-12-29

**Authors:** Li Xiao, Qiannan Hu, Yanshuang Peng, Kaiyue Zheng, Ting Zhang, Lianjie Yang, Zhi Wang, Wanrong Tang, Jie Yu, Qian Xiao, Dandan Zhang, Weifang Zhang, Chanjuan He, Dengxun Wu, Yanyan Zheng, Ying Liu

**Affiliations:** 1 Department of Stomatology North Sichuan Medical College, Afliated Hospital of North Sichuan Medical College, Nanchong, China; 2 Department of Stomatology, Nan Chong Central Hospital, Second Clinical Medical College of North Sichuan Medical College, Nanchong, China; 3 Guanghua School of Stomatology, Guangdong Provincial Key Laboratory of Stomatology, Stomatological Hospital, Sun Yat-Sen University, Guangzhou, China; 4 School of Basic Medical Sciences, North Sichuan Medical College, Nanchong, China

**Correction to: BMC Cancer 21, 1329 (2021)**

**https://doi.org/10.1186/s12885-021-09049-z**

Following publication of the original article [1], the authors noticed that the funding statement was missing. The funding statement is supplied below:

This research was supported by the Pre-research Grant for National Natural Science Foundation of China from North Sichuan Medical College (CBY19-YZ08 to Y. Liu), Clinical and Basic Research Overlapping Fusion Foundation of China from Affiliated Hospital of North Sichuan Medical College (2021LC010 to Y. Liu).
