# Supplementary material for: TRAP1 suppresses oral squamous cell carcinoma progression by reducing oxidative phosphorylation metabolism of Cancer-associated fibroblasts
Source: BMC Cancer. 2021 Dec 14;21:1329. doi: 10.1186/s12885-021-09049-z (PMC8670112; doi:10.1186/s12885-021-09049-z)
Supplement: Supplementary file 1 — Additional file 1: Table S1. Enrichment of cellular components, biological processes, and biological pathways related to mitochondria in differential proteins (CAFs/NFs). [file 12885_2021_9049_MOESM1_ESM.pdf]

Table S1 Enrichment of cellular components, biological processes, and biological pathwaysrelated to mitochondria in differential proteins (CAFs/NFs)

Annotation		No. of genes	No. of genes (database)	Percentage of genes
GOTERM_CC	Mitochondrial nucleoid	*9*	17	52.94%
GOTERM_CC	Mitochondrial membrane	90	566	15.90%
GOTERM_CC	Mitochondrial matrix	15	123	12.20%
GOTERM_CC	Mitochondrial protein complex	14	121	11.57%
GOTERM_CC	Mitochondrial part	28	225	12.44%
GOTERM_BP	Oxidative phosphorylation	7	55	12.73%
GOTERM_BP	ATP synthesis	42	268	15.67%
GOTERM_BP	Electron transport	*15*	159	9.43%
GOTERM_BP	Apoptosis	260	1141	22.79%
GOTERM_BP	autophagy	39	237	16.46%
GOTERM_BP	Oxidative stress	42	204	20.59%
KEGG_PATHWAY	Carbon metabolism	15	60	25%
KEGG_PATHWAY	Parkinson disease	7	46	15.22%
KEGG_PATHWAY	Alzheimer’s disease	8	54	14.81%
KEGG_PATHWAY	Huntington’s disease	9	58	15.52%
