# Supplementary material for: TRAP1 suppresses oral squamous cell carcinoma progression by reducing oxidative phosphorylation metabolism of Cancer-associated fibroblasts
Source: BMC Cancer. 2021 Dec 14;21:1329. doi: 10.1186/s12885-021-09049-z (PMC8670112; doi:10.1186/s12885-021-09049-z)
Supplement: Supplementary file 2 — Additional file 2: Table 2. Identified proteins associated with OXPHOS in different proteins of mitochondria (CAFs/NFs). [file 12885_2021_9049_MOESM2_ESM.pdf]

Table 2 Identified proteins associated with OXPHOS in different proteins of mitochondria (CAFs/NFs)

Protein ID	Gene name	Fold change (CAFs/NFs)	P Value	Subcellular location	Correlation With OXPHOS	DiffStat
P48047	ATP5O	3.87	0.003	Mitochondrion	positive	up
P04406	GAPDH	3.47	<0.001	Cytoplasm	positive	up
P22695	UQCRC2	3.21	<0.001	Mitochondrion	positive	up
P0DMV 9	HSPA1B	2.92	<0.001	Cytoplasm	positive	up
P40939	HADHA	2.73	<0.001	Mitochondrion	positive	up
P21796	VDAC1	2.64	0.001	Mitochondrion	positive	up
P14618	PKM	2.53	0.001	Cytoplasm	positive	up
P60174	TPI1	2.26	<0.001	Cytoplasm	positive	up
P29401	TKT	2.20	<0.001	Cytoplasm	positive	up
P07900	HSP90AA1	2.03	<0.001	Plasma membrane	positive	up
P04075	ALDOA	1.99	<0.001	Cytoplasm	positive	up
P31040	SDHA	1.88	<0.001	Mitochondrion	positive	up
Q8N183	NDUFAF2	1.77	<0.001	Mitochondrion	positive	up
P40227	CCT6A	1.72	0.017	Cytoplasm	positive	up
P55084	HADHB	1.68	0.008	Mitochondrion	positive	up
P36957	DLST	1.56	0.021	Mitochondrion	positive	up
P09972	ALDOC	1.56	0.022	Cytoplasm	positive	up
P10809	HSPD1	1.55	<0.001	Mitochondrion	positive	up
P24752	ACAT1	1.54	0.011	Mitochondrion	positive	up
P49411	TUFM	1.54	0.008	Mitochondrion	positive	up
O75947	ATP5PD	1.52	0.014	Mitochondrion	positive	up
P25705	ATP5F1A	1.52	<0.001	Mitochondrion	positive	up
Q9NVI7	ATAD3A	0.65	0.035	Mitochondrion	positive	down
Q12931	TRAP1	0.5	0.001	Mitochondrion	negative	down
O75390	CS	0.47	0.017	Mitochondrion	positive	down
